# Introducing the Index of Response to Stimulation (IRES): A Novel Metric for Assessing Vagus Nerve Stimulation Outcomes in Drug-Resistant Epilepsy

**DOI:** 10.3390/medicina61010075

**Published:** 2025-01-04

**Authors:** Flavius-Iuliu Urian, Corneliu Toader, Razvan-Adrian Covache Busuioc, Antonio-Daniel Corlatescu, Horia Petre Costin, Gabriel Iacob, Alexadru Vlad Ciurea

**Affiliations:** 1 Department of Neurosurgery, University of Medicine and Pharmacy “Carol Davila”, 030147 Bucharest, Romania; flavius-iuliu.urian@drd.umfcd.ro (F.-I.U.); razvan-adrian.covache-busuioc0720@stud.umfcd.ro (R.-A.C.B.); antonio.corlatescu0920@stud.umfcd.ro (A.-D.C.); horia-petre.costin0720@stud.umfcd.ro (H.P.C.); prof.avciurea@gmail.com (A.V.C.); 2 Neurosurgical Department, University Emergency Hospital Bucharest, 050098 București, Romania; 3 National Institute of Neurovascular Disease, 077160 Bucharest, Romania; 4 Neurosurgery Department, Sanador Clinical Hospital, 010991 Bucharest, Romania; 5 Medical Science Section, Romanian Academy, 050474 Bucharest, Romania

**Keywords:** vagal nerve stimulation, epilepsy, outcome, IRES score, quality of life, seizure reduction, Aspire SR 106

## Abstract

*Background and Objectives*: The Index of Response to Stimulation (IRES) is a new index that we introduce in this study to grade the effectiveness of vagus nerve stimulation in the treatment of drug-resistant epilepsy. We assessed 76 patients at 6, 12, and 18 months after VNS evaluating improvement with the IRES in four key dimensions: seizure duration decrease, seizure intensity decrease, improvement in quality of life, and seizure frequency decrease. This scale goes from 0, meaning no improvement, to 8, meaning maximal improvement, making the scale a really good measure of clinical utility. *Materials and Methods*: This retrospective study followed 76 patients aged 20–65, assessing changes in their IRES scores after VNS therapy using the ASPIRE SR 106 device. Therapy settings were adjusted biweekly to optimize efficacy and patient tolerance. *Results*: There were improvements in the control of the seizures, measured in terms of increased IRES scores. Improvements were associated with quality-of-life enhancements for the patient and a lesser frequency and intensity of the seizures, testifying further to the predictive ability of the IRES toward successful outcomes. This fact reveals that epilepsy treatment must be individual, according to the profile of the patient. *Conclusions*: The study confirms the IRES to be a valid tool for the assessment of the impact of VNS on drug-resistant epilepsy and promotes it as an integral part of the evaluation of the patient for personalized therapy. The findings encourage the use of IRES among the elements that support patient selection and insist on its role in the advancement of precision medicine and optimization of treatment.

## 1. Introduction

In the context of advancing therapeutic approaches for drug-resistant epilepsy, VNS therapy has established a significant role, supported by an extensive body of clinical evidence [1,2]. Outcomes following VNS implantation exhibit considerable variability among patients, necessitating sophisticated monitoring methodologies that encompass a broad spectrum of metrics, including seizure frequency reduction and quality-of-life improvements [3,4,5]. Longitudinal studies have demonstrated that sustained VNS therapy can lead to progressive seizure control, with recruitment time emerging as a critical parameter for assessing therapeutic efficacy [1,6]. Additionally, evidence suggests that VNS outcomes may vary according to factors such as lesional etiology and age at implantation, emphasizing the need for personalized approaches to VNS therapy [2,7].

Recent research highlights the potential role of VNS-induced alterations in heart rhythm complexity as a biomarker, thereby broadening the scope of monitoring techniques [3,8]. While reduction in seizure frequency remains a primary metric, indicating the percentage decrease in seizure occurrence post-treatment, it may not fully capture the multidimensional benefits associated with VNS therapy. Quality-of-life (QoL) scores serve as another vital outcome measure, though these can be influenced by numerous external factors and may lack objectivity.

The array of VNS outcome measures reflects the inherent complexity of drug-resistant epilepsy as a highly individualized condition, thereby necessitating the application of both standardized and emerging metrics—such as seizure frequency reduction and heart rhythm variability—to attain a comprehensive understanding of therapeutic impact [9,10]. Heart rhythm complexity, commonly assessed through heart rate variability (HRV), has shown promise as a biomarker of therapeutic efficacy; however, further validation is warranted to establish its broader applicability. Responder rates, which denote the proportion of patients achieving a predetermined level of improvement, provide a straightforward yet limited metric, as they fail to capture partial responders or improvements in non-seizure-related outcomes [11,12,13].

Studies of VNS cohorts have underscored the therapy’s efficacy beyond seizure reduction, demonstrating improvements in both quality of life and safety profiles, thus supporting its utility as a viable long-term treatment option [5,14]. Moreover, genetic factors underlying epilepsy have been implicated in variable VNS responses, suggesting that genetic analyses should be incorporated into the therapeutic monitoring framework [7,15,16,17,18,19]. These limitations underscore the necessity for a more nuanced and multidimensional outcome measure, leading to the development of the Index of Response to Stimulation (IRES) [1]. This study seeks to validate IRES as an innovative and comprehensive tool for evaluating VNS outcomes, addressing existing gaps in current assessment methodologies.

## 2. Materials and Methods

### 2.1. Study Objective

This study aimed to describe the efficacy of VNS using ASPIRE SR 106 during treatment of drug-resistant epilepsy by using IRES at 6, 12, and 18 months at the University Hospital for Emergency Bucharest (SUUB), from January 2022 to March 2024.

The 76 patients included in the study were all treated at this single center, ensuring a consistent approach to patient follow-up and therapy adjustments. Patients were selected based on their diagnosis of drug-resistant epilepsy and the suitability for VNS therapy as determined by their treating physicians.

### 2.2. Study Design

This was a retrospective study on 76 patients with intractable epilepsy who underwent VNS treatment. We decided to review the medical documents at 6, 12, and 18 months and assign the IRES scores based on patient questionnaires. When patients attended regular follow-ups, they are asked about the frequency, duration, and intensity of seizures and quality of life.

All patients were implanted with the ASPIRE SR 106 device and followed a standardized protocol for VNS settings. Initial therapy settings included an output current of 0.25 mA, with biweekly adjustments to optimize patient tolerance and therapeutic response. This uniform approach ensured that all patients received comparable treatment adjustments, allowing for a consistent evaluation of the IRES scores.

The Index of Response to Stimulation (IRES) was implemented during follow-up visits at 6, 12, and 18 months post-therapy. The tool was administered by trained neurologists specializing in epilepsy care at the University Emergency Hospital Bucharest. The assessment process included delivering standardized interviews and medical record reviews directly to patients and their caregivers where applicable. Patients were evaluated by the attending physician and a neurologist specializing in epilepsy at each follow-up, focusing on emotional well-being, daily functionality, and mood using standardized clinical protocols. While formal mental health tools were not used, assessments were guided by the neurologist’s expertise, supplemented by patient-reported outcomes and caregiver observations.

Inclusion criteria require participants to have drug-resistant epilepsy (DRE) unresponsive to at least two antiepileptic medications, be aged 20 to 65 years, and deemed eligible for VNS therapy by their neurologist. Participants must have undergone implantation of the ASPIRE SR 106 device, be available for follow-up at 6, 12, and 18 months, and provide written informed consent.

Exclusion criteria include coexisting neurological or severe psychiatric disorders, incomplete VNS implantation, or complications leading to therapy discontinuation. Non-compliance with study requirements, pregnancy, breastfeeding, or significant cardiac conditions contraindicating VNS also disqualify participants. Individuals outside the age range of 20 to 65 years are excluded to reduce variability.

### 2.3. Participants

The study population was of 76 individuals aged between 20 and 65 years with drug-resistant epilepsy. The highest frequency was recorded within the range of 30–40 years, as shown below:

Age distribution: 19–29 years, 12 patients (15.79%); 30–40 years, 35 patients (46.05%); above 40 years, 29 patients (38.16%).

Gender: There were 59.21% females, specifically 45 patients, while there were 40.79% males, specifically 31 patients, with a median age for females of 41 years and for males of 32 years.

### 2.4. IRES Score Review

The Index of Response to Stimulation (IRES) score, covering seizure duration decrease (SDD), seizure intensity decrease (SID), improvement in quality of life (IQoF), and seizure frequency decrease (SFD), was the principal outcome measurement in the response to VNS therapy. Scores analyzed from baseline, 6, 12, and 18 months are thus able to provide a more granular timeline of therapeutic results. Seizure duration, intensity, and frequency were assessed through patient-reported diaries corroborated with clinical records and caregiver input when available. Seizure duration was recorded in seconds or minutes per episode, intensity was scored on a visual analog scale (VAS) ranging from 1 to 10, and frequency was determined by the number of episodes per week or month as documented during follow-ups. These metrics were quantified and mapped to the IRES scoring rubric to ensure uniformity in evaluations.

### 2.5. VNS Therapy Settings

The patients were implanted with the ASPIRE SR 106 device at an output current of 0.25 mA after a strategically delayed initiation in the post-operative period, specifically two weeks after surgery to ensure proper healing of the surgical site. Thereafter, device parameters were systematically adjusted every 2 weeks in an attempt to optimize patient tolerance and therapeutic response. The system was advanced by 0.25 mA in the output current on each follow-up adjustment, and other system parameters were fine-tuned with the signal frequency set at 30 Hz, pulse width set at 500 μs, signal on time set at 30 s, and signal off time set at 5 min. The AutoStim was enabled to allow automatic stimulation of the vagus nerve when increased heart rate activity is detected, which usually indicates an impending seizure, providing even more customization of the therapy to the patient’s specific seizure patterns and responses.

### 2.6. Ethical Considerations

All participants in the study gave informed consent for clinical follow-up in respect of ethical guidelines. Such consent ensures that the highest standards of ethical conduct are observed in relation to protecting the rights of patients and that the patient data obtained are maintained confidentially.

### 2.7. Results

A composite metric of IRES score was made for the efficacy of VNS in the treatment of epilepsy. It assessed the therapeutic outcome on four dimensions: SDD, SID, IQoF, and SFD. Consequently, this composite score permits one to draw a balanced and nuanced understanding regarding the impact of the therapy on the patients.

#### 2.7.1. Scoring Criteria

- 0 points means little or no change (0–25% improvement).
- 1 point means minimal to moderate improvement (25–50% improvement).
- 2 points are given to those with strong improvement, more than 50%.
- As each dimension is assessed within this range then the overall IRES score will be from 0 to 8 where 0 represents no observed improvement and 8 represents observed maximum improvement over all assessed aspects.

#### 2.7.2. Interpretation of IRES Scores

- Scores between 0 and 2 indicate minimal or no improvement in the response to VNS therapy and, therefore, the treatment had little effect on the patient.
- Scores of 3–6 are indicative of partial response to treatment in some of the dimensions evaluated, with the improvement being moderate to modest. The range suggests that symptomatic improvement has taken place for the patient following therapy.
- Scores greater than 6 points manifest a highly significant response to treatment or remission, reflecting substantial therapeutic benefit that may be translated into improvement in the quality of life or control over symptoms.

#### 2.7.3. IRES Score Assessment

IRES scores for the patient, evaluated at baseline, 6 months, 12 months, and 18 months, are noted down to provide a full account of stage-wise development of response in the patient under VNS therapy. Long-term follow-up makes it possible to detect progression through various stages and hence offers insight into the effectiveness of the treatment protocol at the various stages. Changes in IRES score will help health professionals to make further decisions regarding the continuation, alteration, or discontinuation of VNS therapy based on the response of a particular patient. Quality of life (QoL) was evaluated by the attending physician by directly questioning the patient based on the score from Table 1. Patients provided responses during follow-up visits, and scores were mapped to the IRES framework, with specific thresholds to differentiate minimal, moderate, and significant improvements.

The QoL score within IRES is comprehensive, encompassing emotional well-being, daily functionality, social interactions, and overall mood. Emotional well-being includes marked reductions in depressive symptoms, which account for part of the score. Daily functionality refers to improving the ability to perform daily activities and maintain independence. Social interactions involve an enhanced ability to engage in social activities and maintain relationships. Overall, mood encompasses general improvements in mood and reductions in anxiety. Each aspect was evaluated, and the QoL score reflects a cumulative assessment. A score of 2 points indicates significant improvements across these areas, not limited to depressive symptoms alone.

**Figure 1:**
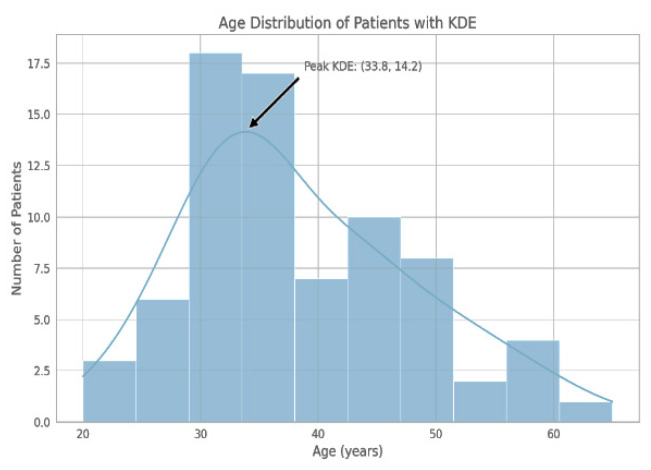
The histogram delineates the frequency of participants by age group, with the largest number of individuals falling within the 30–40 year age range. KDE represents a smoothed density curve, with its mode in the vicinity of 33.8 years.

The demographic distribution of our study cohort showed a notable concentration of individuals in the third and fourth decades of life (see Figure 1).

Out of the 76 patients, 45 (59.21%) were females and 31 (40.79%) were males, making more females than males in the study group. The above findings from the patient demographics serve to underscore the numerical dominance of females in this study, and there is a wider age range among the female patients relative to males.

**Figure 2:**
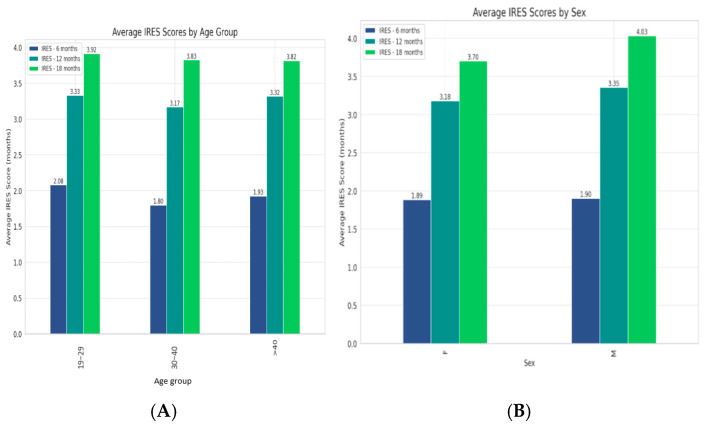
(**A**). Trends in VNS therapy effectiveness across age groups, with all experiencing improvements over time and the 30–40 age group showing the highest average IRES scores. (**B**). Therapy’s efficacy between genders, revealing a slightly stronger long-term response in males.

All age and sex groups experienced an increase in their scores from 6 to 12 months, which either slightly rose or plateaued by 18 months (Figure 2) with a slightly stronger long-term response in males.

The *t*-values and *p*-values are the results of statistical tests comparing the mean IRES scores between two time points. A negative *t*-value reflects that the later *t*-point mean score is higher than the earlier one, reflecting improvement from earlier to later (Table 2).

The mean IRES score progressively increased, rising from 1.88 at 6 months to 3.25 at 12 months and reaching 3.83 by 18 months, indicating an enhanced patient responsiveness to VNS therapy over the treatment period.

**Figure 3:**
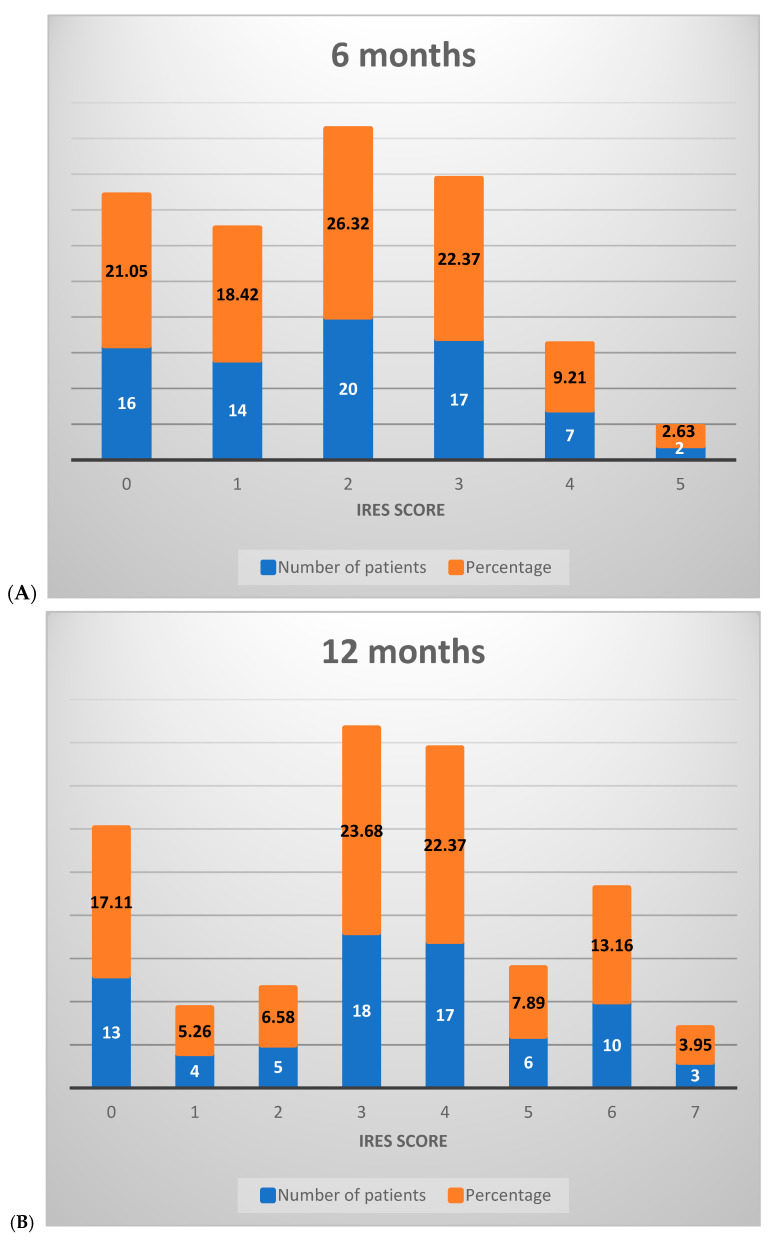

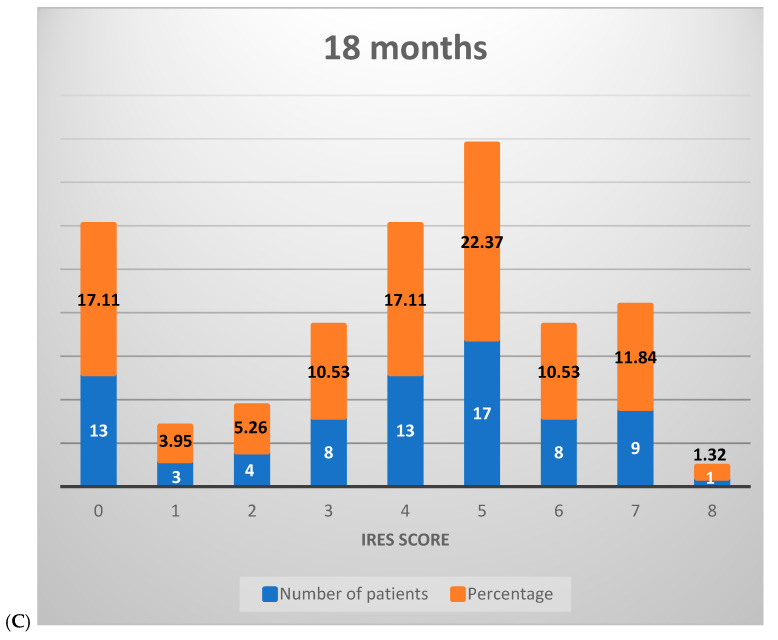
Distribution of IRES scores among patients following VNS therapy: (**A**) 6 months, (**B**) 12 months, (**C**) 18 months.

At the 6-month assessment, the majority of patients (65.79%) scored between 0 and 2 on the IRES scale, reflecting minimal or no therapeutic response, with limited benefits in seizure reduction. A smaller proportion (34.21%) achieved scores between 3 and 6, indicative of a partial therapeutic response. No patients scored between 7 and 8 at this stage, signaling the absence of a substantial treatment effect (Figure 3A).

By the 12-month interval, a notable shift occurred in score distribution: 28.95% of patients remained in the 0–2 range, while a majority (67.11%) scored between 3 and 6, reflecting an improved response across most patients with an elevated partial therapeutic impact. Additionally, 3.95% of patients attained scores between 7 and 8, suggesting a significant therapeutic response in a small subset (Figure 3B).

At the 18-month mark, the distribution further shifted, with 26.32% of patients scoring between 0 and 2, 60.53% between 3 and 6, and 13.16% achieving scores in the 7–8 range. This progression illustrates that, over time, a greater percentage of patients experienced marked improvements, with a growing proportion achieving significant or near-complete therapeutic benefit (Figure 3C).

The descriptive statistics for the IRES score components—seizure duration decrease (SDD), seizure intensity decrease (SID), improvement in quality of life (IQoF), and seizure frequency decrease (SFD)—across the 6-, 12-, and 18-month intervals provide detailed insights into patient responses to VNS therapy for drug-resistant epilepsy:

- *SDD*: shows a gradual increase in mean scores from 0.51 at 6 months to 0.96 at 18 months, indicating a moderate improvement in seizure duration over time.
- *SID:* the mean scores go up from 0.22 at 6 months to 0.55 at 18 months, with a suggestion of slight to moderate reduction in seizure intensity across the study period.
- *IQoF*: marked improvement, with the mean score increasing from 0.74 at 6 months to 1.17 at 18 months.
- *SFD*: the mean scores rise from 0.41 at 6 months to 1.14 at 18 months.

The statistical assurances about the measured improvements over 18 months are given by the confidence intervals (CIs) of IRES components: SFD (0.95, 1.34), SDD (0.78, 1.14), IQoF (1.01, 1.33), and SID (0.40, 0.70). This convergence of the aforesaid parameters into the IRES score, which represents an improvement of significance with a CI of (3.31, 4.35) at 18 months, is a hallmark of the holistic effect of VNS therapy. This multicomponent improvement becomes manifest as the IRES score represents a key parameter to capture the manifold benefits of VNS treatment in drug-resistant epilepsy.

The analysis of Pearson correlation coefficients between the individual components of the IRES score and the overall IRES score demonstrates significant correlations (Table 3).

The strong and statistically significant correlations across all time intervals indicate that improvements in these separate dimensions are consistently associated with improvements in the overall IRES score. Thus, the IRES score is a measure of the multifaceted effects of VNS therapy on the seizure outcomes and quality of life of the patients.

## 3. Discussion

Vagus nerve stimulation (VNS) therapy is one among several interventions for managing drug-resistant epilepsy, with numerous studies documenting various monitoring approaches and therapeutic outcomes in this context [1,2,3,4,5,6,7,8,9,10,11,12,13,14,15,16,17,18,19].

This study validates the use of the Index of Response to Stimulation (IRES) as a robust and comprehensive metric for evaluating the efficacy of VNS therapy in patients with drug-resistant epilepsy. The significant increase in seizure frequency decrease (SFD) scores from 0.41 at 6 months to 1.14 at 18 months underscores the effectiveness of VNS in reducing seizure frequency. This finding is consistent with previous studies that have reported reductions in seizure rates post-VNS therapy. Similarly, the enhancement in improvement in quality of life (IQoF) scores, from 0.74 at 6 months to 1.17 at 18 months, indicates substantial improvements in patient well-being and daily functionality. This aligns with literature demonstrating that VNS not only impacts seizure control but also positively affects overall quality of life.

Furthermore, the increases in seizure duration change (SDC) and improvement in clinical variables (ICV) scores reflect significant reductions in seizure duration and overall clinical improvements. These metrics provide a broader perspective on the therapeutic benefits of VNS, beyond mere frequency reduction. The IRES offers a multidimensional assessment, addressing the limitations of traditional metrics such as seizure frequency alone. By incorporating aspects of quality of life, seizure intensity, and duration, the IRES provides a more holistic view of patient outcomes. The observed increases in SFD scores from 0.41 at 6 months to 1.14 at 18 months indicate a significant decrease in seizure frequency, underscoring the effectiveness of the therapy. This improvement, along with the positive changes in SDD, SID, and IQoF, highlights the comprehensive benefits of VNS therapy as captured by the IRES score.

Although the use of a personalized score such as the IRES provides a nuanced approach to assessing patient progress post-VNS, the overall body of literature seems to indicate a variety of metrics and methodologies available toward ascertaining efficacy for VNS therapy across a variety of populations (Table 4).

Comparing our findings with previous studies, such as those by DeGiorgio et al. [20] and Englot et al. [21], our results reinforce the long-term benefits of VNS therapy. DeGiorgio et al. highlighted reductions in seizure frequency, while Englot et al. emphasized improvements in quality of life and mood. Our study further substantiates these claims, demonstrating significant improvements across multiple dimensions as captured by the IRES. The adoption of IRES in clinical practice can enhance patient monitoring and treatment adjustments, leading to optimized therapeutic outcomes. Future research should focus on further validating the IRES across diverse patient populations and exploring its potential in guiding personalized VNS therapy adjustments. By expanding the discussion to include these critical points and comparative analyses, we provide a more comprehensive interpretation of our findings and their relevance to the field of epilepsy treatment.

## 4. Conclusions

The implementation of the IRES provides a robust framework for assessing VNS therapy outcomes over an 18-month period. The strong correlations between IRES components and the overall score demonstrate the scale’s capacity to detect nuanced effects of VNS on both seizure control and quality of life. This study supports the integration of the Index of Response to Stimulation (IRES) into the suite of tools used for selecting patients for vagus nerve stimulation (VNS). While the IRES primarily evaluates post-stimulation outcomes such as seizure reduction and quality-of-life enhancements, it can also include pre-stimulation assessments to predict potential patient outcomes. By analyzing baseline seizure frequency, initial quality of life, and additional clinical indicators, the IRES aids in identifying candidates likely to benefit most from VNS therapy. This predictive capability establishes the IRES as a valuable tool for both evaluating therapeutic results and selecting suitable candidates, thereby promoting a more tailored and effective treatment approach.

The findings from this study enhance understanding of VNS’s role in epilepsy management and underscore the importance of precision medicine principles. This approach optimizes treatment efficacy and advances the shift toward more individualized healthcare solutions. Overall, this study validates the IRES not only as a comprehensive tool for evaluating VNS therapy outcomes but also as an asset for advancing personalized epilepsy care.

## Figures and Tables

**Table 1:** IRES Score Evaluation for Epilepsy VNS Outcomes.

Component	0 Points	1 Point	2 Points
Seizure Duration Decrease	0–25% improvement	25–50% improvement	>50% improvement
Seizure Intensity Decrease	0–25% improvement	25–50% improvement	>50% improvement
Improvement in Quality of Life	No changes in daily life No observable improvement in the patient’s life after VNS	Feels a little better Reflects a slight but noticeable enhancement in day-to-day well-being	Good improvement Marked reductions in depressive symptoms and an overall betterment of mood and functionality
Seizure Frequency Decrease	0–25% improvement	25–50% improvement	>50% improvement
Total IRES Points:	0–2 points	3–6 points	>6 points
Interpretation:	Minimal or no response	Partial or moderate improvement	Significant or complete response

**Table 2:** IRES mean scores at 6, 12, and 18 months for each age group and sex along with corresponding *t*-values and *p*-values of the significance for changes with time in response to VNS therapy.

Category	IRES 6 Months Mean	IRES 12 Months Mean	IRES 18 Months Mean	6–12 Months *t*-Value	6–12 Months *p*-Value	12–18 Months *t*-Value	12–18 Months *p*-Value
19–29	2.08	3.33	3.92	−5.23945	<0.05	−4.98573	<0.05
30–40	1.80	3.17	3.83	−7.69827	<0.05	−4.99831	<0.05
>40	1.93	3.32	3.82	−7.43701	<0.05	−4.52522	<0.05
Sex F	1.88	3.18	3.70	−9.00464	<0.05	−5.87516	<0.05
Sex M	1.90	3.35	4.03	−7.21193	<0.05	−4.7678	<0.05

**Table 3:** Pearson Correlation Coefficients and Academic Significance Levels between IRES Components and Overall IRES Scores Over 6, 12, and 18 Months.

IRES Score	Pearson 6 Months	Significance *p*-Value 6 Months	Pearson 12 Months	Significance *p*-Value 12 Months	Pearson 18 Months	Significance *p*-Value 18 Months
ICV	0.716	<0.0001	0.863	<0.0001	0.874	<0.0001
SDC	0.779	<0.0001	0.754	<0.0001	0.816	<0.0001
SFD	0.731	<0.0001	0.819	<0.0001	0.834	<0.0001
SIC	0.303	<0.01	0.54	<0.001	0.454	<0.0001

Abbreviations: Improvement in Clinical Variables (ICV): This metric represents the overall improvement in clinical parameters, including seizure intensity and duration. Seizure Duration Change (SDC): Reflects the reduction in the duration of seizures over the study period. Improvement in Seizure Frequency Decrease (SFD): Indicates the reduction in the frequency of seizures experienced by the patient. Seizure Intensity change (SIC): Represents the reduction in the severity or intensity of seizures over the study period, capturing improvements in how debilitating or disruptive individual seizure episodes are for the patient.

**Table 4:** Vagal Nerve Stimulation (VNS) efficacy and outcomes in epilepsy treatment studies.

Study Reference	Patient Cohort	Follow-Up Duration	Main Outcome Measures	Key Findings
Kawai et al., 2017 [1]	385 patients	Up to 36 months	Seizure frequency reduction, quality of life (QoL)	Seizure control progressively improved during treatment, showing a significant median reduction of seizures at various time points.
Colicchio et al., 2012 [2]	53 patients	Mean 55.96 months	Seizure frequency, responder rate, response time (RT)	Lesion etiology and an age at implant of <18 years are found to have a better response.
Liu et al., 2018 [3]	32 patients	1 year	Heart rhythm complexity HRV	VNS reversed the increased complexity in heart rhythm, which might be related to therapeutic effect.
Wang et al., 2019 [4]	Meta-analysis	Varies	Seizure frequency reduction >50%	A significant predictor of VNS outcomes was duration of epilepsy.
Elliott et al., 2011 [5]	436 patients	Up to 11 years	Seizure frequency reduction, safety outcomes	Over 60% of these patients showed a decrease in seizure burden by at least 50%.
Rong et al., 2014 [6]	50 patients	24 weeks	Seizure frequency reduction	The effectiveness and safety of ta-VNS was confirmed with 38% seizure reduction in seizure frequency in patients up to 24 weeks.
Hajtovic et al., 2022 [7]	Meta-analysis on genetic etiologies	Varies	Seizure freedom rate, ≥ 50% seizure reduction	Patients with TSC had substantial seizure reduction; those with DS had less robust seizure reduction.
Constantinescu et al., 2020 [8]	5 patients	3 months	HRV parameters	VNS did not alter cardiac autonomic function after 3 months of neurostimulation.
Sen et al., 2021 [9]	International registry	36–60 months	Seizure frequency, seizure severity, QoL	Aims to examine long-term safety and clinical outcomes of VNS in people with DRE.
Liu et al., 2018 [10]	63 patients	1 year	Preoperative HRV, VNS outcomes	Preoperative HRV analyses can help predict VNS outcomes in patients with DRE.
Bauer et al., 2016 [11]	Randomized trial	20 weeks	Seizure frequency reduction	No superiority of 25 Hz tVNS over 1 Hz tVNS in reducing seizure frequency.
Zhang et al., 2019 [12]	110 patients	Post-operative period	Seizure frequency and severity, McHugh seizure outcome	Significant reduction in seizure frequency observed; VNS effect improves over time.
Lagae et al., 2015 [13]	70 patients	Varies	Responder rate, age at implantation effect	Younger age at VNS implantation might result in a better outcome.
Benedetti-Isaac et al., 2012 [14]	4 patients	Post-VNS implant	Seizure frequency reduction	VNS was an alternative for seizure frequency reduction in patients with previous corpus callosotomy.
Nalbantoğlu et al., 2014 [15]	35 patients	26 ± 19.2 months	Seizure outcomes, responder rate	A total of 80% of the patients were responders, thus showing VNS as an alternative good treatment.
Verrier et al., 2016 [16]	28 patients	Pre- and post-implantation	T-wave alternans (TWA) levels, cardiac electrical stability	TWA level was decreased by VNS treatment in 70% of the patients.
Shan et al., 2022 [17]	45 patients	November 2016–August 2021	Clinical outcome, safety	Confirmed efficacy and safety of VNS; no significant prognostic factors were identified.
Bao et al., 2011 [18]	45 cases	Over 1 year	Seizure frequency, treatment duration	Critical for improved prognosis was a longer duration of VNS therapy.
Tanaka et al., 2017 [19]	Randomized, double-blind clinical trial	20 weeks	Efficacy and safety of tVNS	tVNS was associated with high adherence to treatment and was well tolerated. The efficacy results justify further trials.

## Data Availability

All data is available on reasonable request to the corresponding author.

## Abbreviations

CI = confidence interval; DRE = drug-resistant epilepsy; DS = Dravet syndrome; HRV = heart rate variability; IRES = Index of Response to Stimulation; IQoF = improvement in quality of life; QoL = quality of life; SDD = seizure duration decrease; SID = seizure intensity decrease; SFD = seizure frequency decrease; ta-VNS = transauricular vagal nerve stimulation; TSC = tuberous sclerosis complex; TWA = t-wave alternans; VNS = vagal nerve stimulation.
